# On the Treatment of Puerperal Mania
*Read at Med. Society of London, Dec. 2, 1854.


**Published:** 1855-04-01

**Authors:** J. M. Winn

**Affiliations:** Licentiate of the Royal College of Physicians, &c. &c.


### ON THE TREATMENT OF PUERPERAL MANIA.*

BY J. M. WINN, M.D.,

*Licentiate of the Royal College of Physicians, &c. &c.*

The prevalence of opinions, with regard to the moral management of patients suffering from puerperal mania, which I hold to be, not only erroneous, but dangerous, has induced me to choose the treatment of this disease as the subject of discussion to-night, in the hope that it may be the means of eliciting the views of the fellows of this Society on some points of the highest practical importance.

It would be impossible in the short compass of time allotted for the reading of this paper to enter fully upon all the points connected with the treatment of puerperal insanity, I shall therefore be compelled to pass briefly over that division of the subject which relates to the physical treatment of the disease, in order that I may dwell at more length on its *moral* management, a question of far greater importance, and on which the successful termination of a case more especially depends.

Insanity may attack a patient at three periods during the puerperal state—1st, during gestation; 2nd, subsequent to delivery; and 3rd, during lactation, in consequence of protracted suckling. The first form for the most part is a transient affection, and generally disappears on the termination of labour. The third variety of the disease generally manifests itself as melancholia, and depends on an anæmic condition of the system. The remarks which I am about to offer have especial reference to the second form of the malady, to that maniacal excitement which supervenes during the first months after delivery, and appears to be the result of extreme irritability of the brain, associated with great nervous exhaustion.

Before entering upon the immediate subject of my paper I think it necessary to object to the term of puerperal mania, used as it commonly is in a *specific* sense, and the use of which term has led, in my opinion, to serious errors in practice. When adopted as an expression of a mere variety of insanity, it is sufficiently distinctive and appropriate; but a serious mistake is involved in the supposition that the disease, commonly termed puerperal mania, is a *special* form of insanity, requiring a treatment entirely different from that which is laid down for the cure of mania in general. No doubt the peculiar condition of the blood, and the excitable state of the nervous system which obtain after delivery, tend to modify a maniacal attack, and to render it necessary that the treatment should be adapted to these qualifying circumstances. Nevertheless, the affection is essentially mania, and must be treated as such.

On reviewing the symptoms of puerperal insanity, it will be readily seen that the features of this disease are perfectly identical with those of an ordinary attack of mania, and for this purpose I refer to a sketch of the symptoms of puerperal mania which I published in my "Manual of Midwifery." This sketch is brief but true to nature, and quite sufficient to establish the truth of my assertion.

"It" (puerperal mania) "may come on suddenly, but its accession is often marked by premonitory symptoms. The earliest indications are restlessness, an anxious expression of countenance, peevishness, slight incoherence, and extreme talkativeness. Sometimes there is an opposite condition in which the patient is taciturn and listless. As the disease advances all the symptoms become aggravated, and the patient's mind is occupied with various delusions. She often expresses a hatred towards her husband and child, and frequently utters oaths and obscene language. A tendency to suicide is very common,

* Read at Med. Society of London, Dec. 2, 1854.

and the persistence of extreme restlessness is often one of the most inveterate symptoms. Sleeplessness will often continue for nights together, and resist the influence of the most powerful narcotics."

The importance of this generalization will be more clearly shown when I come to consider the expediency of removing puerperal lunatics to an institution especially set apart for the care and cure of the insane.

Before entering upon the treatment of puerperal mania, it is absolutely essential to determine, as far as the present state of mental pathology will permit, that particular condition of the system which gives rise to the distressing malady in question. Frequent and careful clinical observation has led me to infer that puerperal mania is the result of cerebral irritation combined with great nervous exhaustion, a deteriorated condition of the blood, and an imperfect nutrition of the nervous tissue. Granting these facts, it follows that an antiphlogistic mode of treatment is decidedly objectionable, and that the classes of remedies most likely to prove beneficial are sedatives, depuratives, tonics; some leeches may occasionally be employed to allay irritation of the brain, but on no account as a depleting measure. Venesection and blistering are positively injurious. A nourishing, though not a stimulating diet, is generally required; whilst opiates, gentle aperients, and diuretics, will all be found more or less useful. It is, however, on the moral treatment of a case that the recovery chiefly, if not wholly, depends. The psychical management of the patient is precisely the same as that which is generally indicated in *any* variety of mania. The first and most important part of the treatment is to provide an efficient nurse capable of maintaining a kind but firm control over the person committed to her charge. The patient must be incessantly watched. The fireplace should be guarded, the windows firmly fastened, and every other precaution adopted by which the danger of self injury may be obviated. The greatest tranquillity must also be observed, and the patient's friends and relatives rigorously removed from her presence. The great object is to break the morbid current of thought, by a seclusion more or less complete, and thus give rise to emotions and ideas entirely new. The advantage of thoroughly changing the association of ideas, is clearly proved by an interesting fact which Esquirol mentions: he observed that recoveries took place much sooner, and more frequently amongst those patients who came to Paris to be under his care, than amongst those who were inhabitants of that city.

The most important point, however, in connexion with the moral treatment, and one which deserves the most earnest consideration, is the question of the patient's removal to an asylum, when the ordinary remedies have failed to afford relief.

However inexpedient and culpable it would be to hurry a patient to an asylum at the outset of puerperal or any other form of mania, still a period may arrive, and that within very few weeks after an attack, when delay in having recourse to this measure, will subject the patient's health and life to the greatest danger. I regret to find that general opinion is either entirely opposed to the removal of a case of puerperal mania to an asylum, or at best, only sanctions this step as a last resource, when, as too often happens, the curable stage has irretrievably passed away. To combat a doctrine, fraught with such imminent peril, is the principal object of my present communication.

Dr. Forbes Winslow has clearly established the fact, in his treatise on the "Incubation of Insanity," that it is only during the earlier periods of an attack of mania that a cure can be expected from the use of appropriate remedies.

By a strange perversion of a general principle, this important truth has been very commonly lost sight of in the moral treatment of puerperal mania. In evidence of the frequency or this dangerous error, I have selected the opinions of some well-known modern writers, which will be amply sufficient to show that I have by no means over-rated the amount of the evil in question. I have not quoted the names of the authors, fearing it might seem invidious to select the names of some few individuals, whilst there are so many of our profession who entertain views precisely similar.

One deservedly high authority says:—"Removal to an asylum is not so frequently requisite for the mental disorders of puerperal patients as for insanity occurring in other circumstances. It is principally required in the more obstinate and prolonged cases; and after other measures of partial or complete seclusion have been tried." Another author observes:—"I well know that patients labouring under puerperal insanity have sometimes been sent to lunatic asylums. Such a step, in such circumstances, is so inconsistent with every feeling prevailing in social life that whenever it is taken the whole responsibility and the whole odium of it must rest with the medical adviser."

Another writer makes the following highly reprehensible remark:—"These cases (puerperal mania) ought not to be sent to a *mad-house*, it being very rare for puerperal mania to continue long, especially when early and properly treated."

Before I proceed to contravene the opinions of these writers, so dogmatically asserted, I must protest most earnestly against the *term mad-house* employed by the author whom I have last mentioned. It is the duty of medical men to point out the advantage of such institutions as our asylums for the insane, rather than to excite the prejudice of unthinking minds against these valuable establishments. Appropriate enough as this term might be for those asylums of the darker ages when every description of cruel restraint was practised, it is singularly inapplicable now, when humanity and science are alike enlisted to allay the sufferings and ameliorate the condition of their inmates. It is to be hoped that before many years have elapsed, even the milder term of lunatic asylum will be abolished, and the far more appropriate title of "Hospital for the *cure* of the Insane," as recommended by Dr. Winslow, will be generally adopted.

About two years since, I was requested to see, in consultation with Mr. Meeres, a baker's wife, æt. 37, residing in Brick-lane, who had been attacked with mania five weeks after the delivery of her seventh child. I found her in a great state of excitement, talking incessantly, and her mind filled with phantasms. Her chief delusion consisted in the belief that she was suffering from poison which had been administered to her before my arrival. The tongue was white; the countenance animated and cheerful; and the body not in the least degree emaciated. Her watchfulness was incessant. Perfect quietude, a full dose of morphia, with as much seclusion as her circumstances would permit, were prescribed. This course was followed during several days, but without avail; neither sleep nor tranquillity were to be obtained. As this patient lived in the confined apartment of a small house, and was unable to procure the attention absolutely necessary for her recovery, I advised her immediate removal to an asylum, which was done a week after the commencement of her attack. In three months she was restored to her family perfectly well. Since then she has been confined with another child without any recurrence of the mania. It is right to add that this patient was naturally of a very excitable temperament, although there was not the slightest hereditary tendency to mania in her family.

I have selected this instance of perfect recovery from my note-book as one that strikingly illustrates the advantage of sending suitable cases to an asylum. In not one instance have I seen any ill consequences follow the prompt transfer of these cases; so far from it, I have frequently had to deplore the evils arising from delay. One case, especially, that I attended in the neighbourhood of the Regent's-park, recurs most forcibly to my recollection as illustrative of the dangers of procrastination. This patient had been ill for many months, whilst all the routine practice had been employed in vain. Change of air and scene had been tried in vain. I cannot help believing that the long and painful probation which her family had to endure might have been spared them had she been removed to a well-regulated institution at an early period of her disorder.

Some interesting statistical returns of Bethlem Hospital, referred to by Dr. Webster in a valuable paper which he read before this Society several years since, afford incontestable proof that the treatment of puerperal mania, as conducted in large asylums, is pre-eminently successful. Dr. Webster observes—"As to the curability of this form of mania, nine recoveries more were reported than in the other varieties of lunacy; eighty-one puerperal patients having been cured, or at the rate of 61·83 per cent., whereas the average recoveries during the last twenty years in all cases of insane females treated at this institution was 53·67 per hundred. Hence three in every five cases of puerperal insanity may be confidently expected to recover within a year.

My friend, Dr. Theodore Boisragon, the medical superintendent of the Cornwall County Lunatic Asylum, has obligingly favoured me with the following information, strongly corroborative of the opinion I have advanced. He says, in reference to his experience of puerperal mania at various asylums—"The majority of cases recover; in fact, I do not remember having discharged a single case that was not convalescent. I perfectly coincide with you as to the propriety of sending these cases to an asylum. It is of course perfectly clear that they are for the time decidedly insane, and need that description of treatment for which the structure and appliances of an asylum are peculiarly adapted. When at Warwick I had charge of a case which affords a good illustration of this assertion. A private patient was treated at her own home by a distinguished physician of that town; but his advice was completely frustrated by the injudicious interference of the patient's mother. The consequence was that over indulgence was resorted to at one time and mechanical restraint at another, so that the patient might have become incurably insane, had not her husband insisted on placing her under my care after the disease had persisted for thirteen weeks. The means employed were quietude, seclusion, and amusement. The result was very striking: at the end of thirteen weeks more she was discharged convalescent, and her ultimate recovery was perfect."

The preceding facts must convince every unprejudiced mind, that with regard to the treatment of puerperal mania, the removal to an asylum, so far from proving prejudicial, is attended, on the contrary, with the happiest results to the patient, and cannot be too strongly advocated.

There are some, however, who admit the efficacy of this plan of treatment, but disapprove of it on the ground that the mere recollection of having been confined in an asylum will produce an injurious effect on the mind of the patient in a subsequent labour. I have never noticed such a consequence, and in the case to which reference has already been made, as occurring in my private practice, the patient's subsequent labour was unattended with the slightest mental aberration.

Another common prejudice which has not unfrequently interfered with the timely removal of a patient to a lunatic asylum, is the dread entertained by the friends of the individual lest some sort of opprobrium be incurred by her having been the inmate of what is vulgarly called a mad-house. This prejudice, which arises either from ignorance or mistaken delicacy, is rapidly passing away. Did the knowledge of Robert Hall's and Cowper's insanity diminish, in the slightest degree, the affectionate admiration in which these highly-gifted individuals were held? Did it not rather tend to increase public sympathy and private regard? The supposition that puerperal mania is generally a very transient affection is another erroneous opinion, which has frequently interfered with the early removal of patients.

The yearly records of our lunatic asylums furnish many examples of the chronic and protracted form of this disease arising entirely from the early neglect of appropriate measures.

In conclusion, I wish it to be clearly understood, I do not recommend the hasty and indiscriminate removal of a patient. There are many cases that may be cured without this measure being adopted. At the present time I have a young lady under my care, whose aberration of mind arises from derangement of the uterine functions, but who is rapidly recovering under the influence of a treatment, which has chiefly for its object the removal of the physical disorder. This lady had been ill for a considerable period before she came under my care; she had tried change of air and scene, with a variety of remedies; but the somatic treatment having proved so strikingly successful, I have not deemed it necessary to place her under surveillance.

In the humbler walks of life, cases of insanity should be generally removed at an early period to an asylum. For rich patients this measure may not always be necessary; they can obtain a quiet residence, all the comforts, and many of the advantages of an asylum. Not so with the poor. Confined, perhaps, to a close room, in a narrow and noisy street, insufficiently nourished and badly nursed, the poor patient is cut off from all hope of a cure. For cases of this description an asylum offers the only chance of cure.

---

